# Supplementary material for: PINK1/Parkin-dependent mitophagy mediates astrocytic inflammatory responses to mitochondrial damage
Source: bioRxiv. 2026 May 13:2026.05.11.724378. Preprint. [Version 1] doi: 10.64898/2026.05.11.724378 (PMC13192790; doi:10.64898/2026.05.11.724378)
Supplement: 1 [file NIHPP2026.05.11.724378V1-supplement-1.pdf]

bioRxiv preprint doi: https://doi.org/10.64898/2026.05.11.724378; this version posted May 13, 2026. The copyright holder for this preprint (which was not certified by peer review) is the author/funder, who has granted bioRxiv a license to display the preprint in perpetuity. It is made available under a CC-BY 4.0 International license.

Supplementary Table 1. Raw concentrations of secreted factors (pg/mL) detected in media following treatment with 5 μM BMS345541 or vehicle control for 72h, as well as OXPHOS inhibitors (10 μM Antimycin A, 5 μM Oligomycin A) or vehicle control for the final 16h of treatment.

Cytokine	Control	AA/OA	AAOA+BMS345541
6Ckine_Exodus_2	5.861667	5.633333	2.608333
EPO	11.85	10.12667	8.16
Eotaxin	2.101667	0.95	0.656
Fractalkine	53.8975	56.78	51.37833
GM_CSF	47.28667	34.73167	6.083333
G_CSF	621.8933	252.988	5.728
IFNb_1	60.96	77.98167	29.76
IFNg	3.133333	1.678	0.73
IL-10	1.716	3.15	
IL-11	2.958333	7.306	1.803333
IL-12p40	8.738	6.696667	
IL-12p70	9.438	10.31167	2.986
IL-13	2.52	1.6275	0.11
IL-15	5.91	1.843333	4.2475
IL-16	5.673333	5.27	3.901667
IL-17	2.145	2.053333	0.685
IL-1a	23.45167	20.15667	7.172
IL-1b	25.284	25.562	2.205
IL-2	1.425	1.101667	0.646667
IL-20	14.04833	9.675	8.525
IL-3	0.753333	0.613333	0.52
IL-4	0.236667	0.236667	0.18
IL-5	0.458333	0.49	0.288333
IL-6	3507.245	2989.228	44.774
IL-7	1.141667	0.942	0.914
IL-9	13.815	12.27833	7.94
IP_10	2491.642	2440.234	938.7167
KC	4994.953	3663.283	513.096
LIF	9.505	45.265	8.672
LIX	51.178	74.005	27.3175
MCP_1	5805.165	5695.426	2037.457
MCP_5	9906.857	2935.898	266.688
MDC	43.246	9.27	1.636
MIG	1.081667	0.726	0.235
MIP_1a	4438.593	3480.05	338.972
MIP_1b	6443.92	5567.52	771.744
MIP_2	4756.318	3325.183	180.228
MIP_3a	33.21333	24.555	8.681667
MIP_3b	26.802	22.84833	12.49167
M_CSF	2.133333	1.436667	0.703333
RANTES	483.1817	437.8567	43.696
TARC	7.025	6.913333	1.268333
TNF_a	499.502	667.626	18.49
VEGF	18.902	39.17333	32.86
